# Management of Japanese Encephalitis: A Current Update

**DOI:** 10.7759/cureus.14579

**Published:** 2021-04-20

**Authors:** Abimbola O Ajibowo, Juan Fernando Ortiz, Ammar Alli, Taras Halan, Olasunkanmi A Kolawole

**Affiliations:** 1 Internal Medicine, Fort Worth Renal Group, Dallas, USA; 2 Neurology, Larkin Community Hospital, Miami, USA; 3 Neurology, Universidad San Francisco de Quito, Quito, ECU; 4 Medicine, Tishreen University Faculty of Medicine, Lattakia, SYR; 5 Internal Medicine, Universitat de Barcelona, Barcelona, ESP; 6 General Medicine, Ternopil National Medical University, Ternopil, UKR; 7 Internal Medicine, University of Texas Southwestern Medical Center, Texas, USA

**Keywords:** japanese encephalitis

## Abstract

Japanese encephalitis (JE) continues to be one of the world’s most serious infections with no definitive treatment or guidelines. The high morbidity and mortality rate among symptomatic patients warrant the need for further investigation in this regard. Our review focuses on the recent updates on Japanese encephalitis treatment. For that reason, we used an advanced PubMed search with JE and drugs like minocycline, interferon, ribavirin, immunoglobulin, dexamethasone, and acyclovir. All research was done in full papers written in the English language and conducted in humans. This review aims to compare and analyze recent papers regarding JE treatment to guide healthcare providers with the latest information and make evidence-based decisions when presented with this infection. Overall, only minocycline had promising results because one of the two studies showed statistically significant results. The second study showed positive trends in children over 12 years and patients who survived on the first day of hospitalization. The study with intravenous immunoglobulin (IVIG) did not improve the outcomes; however, it increased the levels of neutralizing antibodies. Further study with higher doses may change the outcomes in patients with JE. The other drugs failed to show promising results.

## Introduction and background

Japanese encephalitis (JE) is a flavivirus [1]. The endemic encephalitis viral disease mostly occurs in Eastern and Southeastern Asia [2]. The disease is caused by a positive-stranded ribonucleic acid (RNA) virus surrounded by a 50 nm glycoprotein and enclosed in a nucleocapsid envelope. Fifty percent (50%) of survivors have a neurological disability [2-3]. The virus is closely related to West Nile Virus (WNV).

Humans contract JE in case of a bite from an infected mosquito [4]. After penetration in the skin, the virus replicates in Langerhans dendritic cells or keratinocytes to be carried to the local lymph node for additional replication [1]. Once replication completes, the virus amplifies to produce viremia, crosses the blood-brain barrier to enter the central nervous system, causing a diffuse brain infection or encephalitis in some cases [5].

Every year, JE infects more than 50,000 people annually. The ratio of symptomatic to asymptomatic cases is 1:300 [2]. The disease affects children more than adults with a ratio of 200:1; The infection is more prevalent in June and July [3]. The infection has a 10%-30% fatality rate, and 30%-50% of those patients develop neurologic sequela [3].

The symptoms usually begin with coryza, diarrhea, headaches, and rigors. These symptoms are then followed by alteration in the level of consciousness and seizures [6]. A significant complication of the disease is deafness, flaccid paralysis, movement disorders such as Parkinsonism, dystonia, and hemiballismus. The involvement of the anterior horn causes flaccid paralysis [1,6].

Physicians should verify clinical symptoms and confirm destinations of recent visits. If a patient traveled in PE endemic regions, they would undergo a cranial CT or an MRI scan of the brain to check for any abnormalities [5]. The most common places with abnormal signals in descending order are the thalamus (94%), mid-brain (58%), basal ganglia (35,5%), cerebellum (25,8%), pons (19%), and cerebral cortex (19%). When a patient has clinical features of encephalitis and bilateral thalamic abnormalities on MRI, a diagnosis of JE is very suggestive [7]. Most commonly, physicians detected immunoglobulin M (IgM) in serum or cerebrospinal fluid with an enzyme-linked immunosorbent assay (ELISA) test [1].

Table 1 summarizes the epidemiology, pathophysiology, clinical features, diagnosis, and MRI findings of JE [1-7].

**Table 1:** Clinical features of Japanese encephalitis MRI: magnetic resonance imaging; CSF: cerebrospinal fluid

Epidemiology, pathophysiology, clinical Manifestations, and diagnostics	
Epidemiology	Eastern and Southeastern Asia infects more than 50,000 people and causes about 15,000; the disease affects children more than adults with a ratio of 200:1; the infection is more prevalent in June and July.
Clinical Manifestations	Vomiting, nausea, headache, high fever, stiff neck, spastic paralysis, and tremors. Patients with encephalitis have distortion of consciousness and convulsions.
Complications	Movement disorders, deafness, flaccid paralysis, spasticity.
Diagnostics	IgM in the serum of CSF (more specific), with ELISA test. Antibodies are usually detectable between 3-8 days after the onset of illness and may persist for several months. EEG may show alpha, delta waves.
MRI findings	MRI reveals a prominent hypointense lesion on T1 and hyperintense on T2 in the sub-acute stage suggestive of hemorrhagic changes in the thalamus, basal ganglia, substantia nigra, cerebellum, pons, cerebral cortex, and spinal cord.
CSF findings	Cells: moderate lymphocytic pleocytosis; Protein: high; Glucose: normal; Pressure: normal/high

Luckily, the incidence of the infection has diminished in recent years due to vaccines, especially in Asian countries [2]. However, physicians must be prepared and updated when treating patients with JE. We conducted a review of the main drugs used in JE to consolidate this infection's management. JE in non-endemic areas has occurred, and vaccines are not recommended in non-endemic areas. That is why doctors should be prepared with the best treatments to treat JE [8].

## Review

Methods

According to Turtle et al., five drugs have been used in clinical trials for JE: minocycline, interferon, ribavirin, immunoglobulin, and dexamethasone [1]. We used a PubMed advance search strategy to find additional papers that support the results of the clinical trials. Additionally, we searched papers related to acyclovir use in JE because acyclovir is commonly used in viral encephalitis [9]. Table 2 shows the search results for each drug.

**Table 2:** Methods of the study

Drug	Total records extracted
(steroids [Title/Abstract]) AND (Japanese encephalitis [Title/Abstract]) OR (dexamethasone [Title/Abstract]) AND (Japanese encephalitis [Title/Abstract])	10
(Japanese encephalitis [Title/Abstract]) AND (ribavirin [Title/Abstract])	5
(Japanese encephalitis [Title/Abstract]) AND (Minocycline [Title/Abstract])	13
(Japanese encephalitis [Title/Abstract]) AND (acyclovir [Title/Abstract])	9
(Japanese encephalitis [Title/Abstract]) AND (Interferon alpha [Title/Abstract]) OR (Japanese encephalitis [Title/Abstract]) AND (Interferon alfa [Title/Abstract])	18
(intravenous immunoglobulin [Title/Abstract]) AND (Japanese encephalitis [Title/Abstract])	7

Results

**Figure 1:**
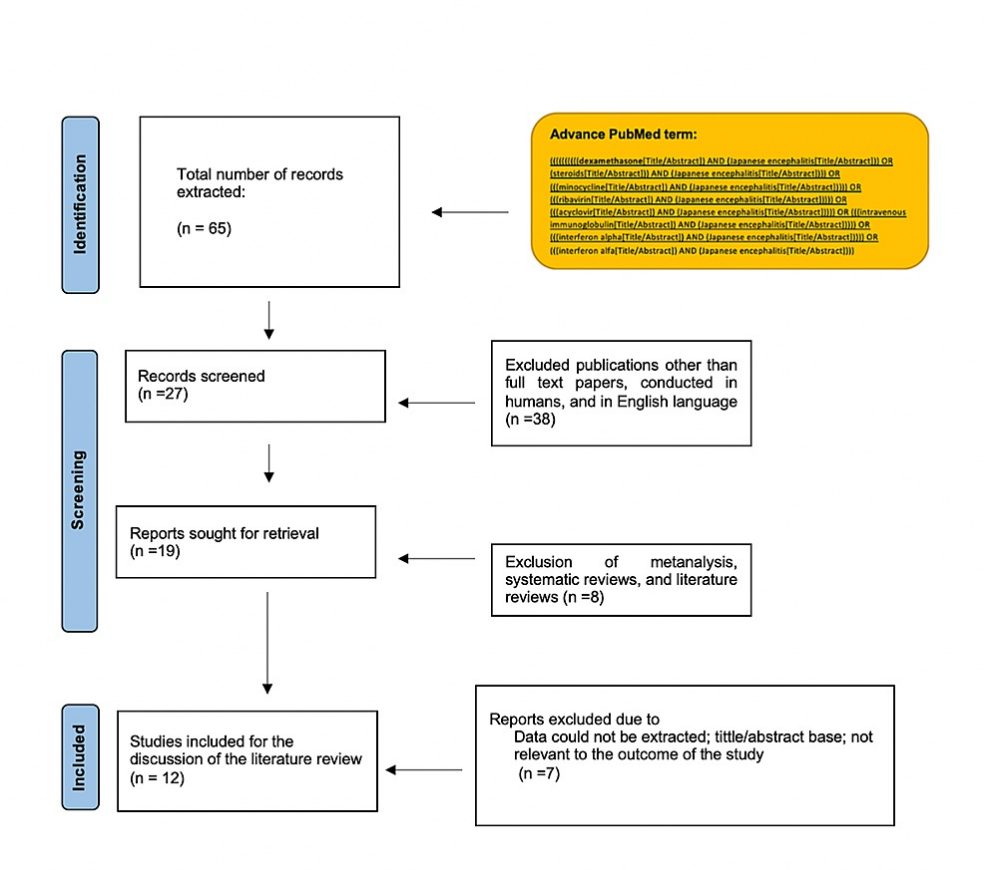
Results of the study

For the discussion of the literature review, we only included full-text studies of humans written in the English language. We excluded systematic reviews, literature reviews, and meta-analyses. Figure 1 shows the step-by-step results of the study.

Table 3 shows the methods, outcomes, and limitations of the studies used for this paper [5-7,10-18].

**Table 3:** Characteristics of the studies IVIG: intravenous immunoglobulin; CNS: central nervous system

Author, year, country	Methods	Intervention	Outcomes	Limitations
Hoke et al., 1992, Thailand, [5]	Randomized double-blind placebo control trial. Twenty-five patients received dexamethasone, while 30 patients received a placebo.	Dexamethasone.	There was no clinical benefit or detriment with the use of dexamethasone. Mortality did not vary among the placebo or control group.	The power of the study was small: ( β = 0.5). A study with a higher number of participants may show clinical benefit.
Johnson et al., 1986, Thailand, [10]	Retrospective study, during an outbreak in North Thailand. They compared the death rate of six patients who received dexamethasone with seven patients who did not.	Dexamethasone	There was no difference in the mortality rate in both groups.	Small sample, but no major flaws.
Rathi et al, 1986, India, [11]	Comparative study: 137 out of 875 patients received dexamethasone + dopamine during an outbroke in India.	Dexamethasone and dopamine	Mortality or hospital stay was no statistical difference in patients.	The risk of bias in this study appeared to be low.
Kumar et al., 2016, India, [12]	Randomized double-blind placebo-control trial. 140 patients received the drug, while 140 received a placebo.	Minocycline	There was no statistical difference in mortality among the two groups. However, there was a positive trend. Overall, the outcome of GOS at three months was close to being statistically significant p = 0 · 059. There was a positive trend in children above 12 years and patients who survived their first day of hospitalization.	A variable number of doses was received by the patients. It was not measured the level of minocycline in the CNS.
Singh et al., 2016, India, [13]	Randomized placebo-controlled trial. 44 patients received Minocycline while 50 patients received placebo.	Minocycline	The duration of fever, duration of hospitalization, and unconsciousness were statistically significantly reduced in the treatment group. However, mortality and prevalence of neurological deficits after 12 months did not vary among both groups.	The study sample was small, and the author suggests a larger follow period in future studies to determine the drug's clinical benefit.
Kumar et al., 2009, India, [14]	A randomized, double-blind placebo-controlled trial. In the study, 70 patients received ribavirin and 87 received placebo; The author used an intention-to-treat analysis.	Ribavirin	There was no statistically significant difference between both groups in early mortality and late outcomes.	Lack of response to ribavirin could be related to the small study sample and because the drug may not have given early enough.
Zhao et al, 2020, China, [7]	Case report	Ribavirin	The patient received ribavirin, and his symptoms improved. After six months, he continued with Parkinsonian features.	No limitations are described or found.
Rayamajhi et al., 2015, Nepal, [15]	22 children participated in the study. Eleven children received IVIG and 11 received placebo.	IVIG	Mortality or clinical outcomes did not differ between the two groups. However, in the treatment group, the neutralizing antibody titers, IL-4, and IL-6 were higher than in the control group.	The sample of the stud was small. There was not a satisfactory explanation for the increase in antibody response and cytokines in the treatment group.
Soloman et al. 2003, Vietnam, [16]	Randomized double-blind placebo-controlled trial. The authors made an intention to treat analysis. IVIG was given to 61 children and placebo to 56 kids.	Interferon	There was no difference in hospital deaths or severe sequelae at discharge. Outcome at three months of being discharge did not differ between both groups.	Small study sample. The risk of bias was low.
Harinasuta et al., 1985, Thailand, [17]	Case Series: Two patients received interferon-alpha, while the other two did not receive interferon	Interferon	Both patients have good clinical outcomes. The first patient started improving on day five and continued improving slowly. The second patient recover from a comatose stage on the 6th day; at two weeks, he had complete recovery without neurological sequelae The patients who did not receive interferon die on days 7th and 9th of hospitalization.	No limitations are discussed or found
Shen et al., 2020, China, [6]	Case Report	Acyclovir	A 30-year-old man with flaccid paralysis due to Japanese encephalitis was treated 12 days with acyclovir without any improvement. He did not die but developed neurological sequelae.	No limitations are discussed or found
Ayukawa et al., 2002, Japan, [18]	Comparative study: Six patients were treated with Acyclovir during an outbreak in Japan. There was not a control group	Acyclovir	Four patients have severe neurological sequela. One patient died, and one patient recovered without sequelae.	There was not a control or placebo group. The sample was too small.

Discussion

Dexamethasone

Dexamethasone use in JE has been reported in a clinical trial and two outbreaks, one in North Thailand and the second in India.

Patients died five days after having been hospitalized with JE, due to coma or respiratory arrest [5]. Given this fact, there was a rationale that steroids may reduce brain edema in these patients. A clinical trial with dexamethasone did not show statistical differences between the treatment group and placebo in the clinical control trial [5]. There was no benefit in mortality, number of days hospitalized, number of days with ventilator, or number of days with mannitol.

During an outbreak in North Thailand, 13 patients were diagnosed with Japanese encephalitis. Six of these patients were treated with dexamethasone while seven did not receive dexamethasone. Two patients treated with steroids died, and four patients treated without steroids died, showing a better outcome in patients with steroids. However, a sample of 13 patients is too small to draw conclusions [10]. Pleocytosis remarkably diminished in the treatment group as compared to the treatment group. The treatment group has a mean of 152 and 92 cell/ mm3 on Days 4 and 5 compared to the untreated group with 321 and 280 cell/mm^3^. There was no change in the percentage of lymphocytes [10].

In 1988, there was an epidemic in Gorakhpur, India; 137 patients out of 875 received dexamethasone and dopamine. Overall, dexamethasone did not improve the patients' clinical outcome compared to patients who do not receive dexamethasone. The deaths occurred mainly in the acute phase of infection, around three days after admission. There was no statistical significance between both groups in terms of mortality or days of hospitalization [11].

Overall, dexamethasone did not appear to be beneficial to patients with JE.

Minocycline

Minocycline is a semi-synthetic tetracycline shown to be effective in both animal and human models of JE. Minocycline penetrates the brain-blood barrier due to its lipophilic nature. The drug also inhibits the ribosome subunit 30s in bacterias [12]. Two clinical trials have been conducted evaluating the efficacy of minocycline in Japanese encephalitis [12-13].

A controlled clinical trial by Kumar et al. involving 281 patients studied mortality at three months of hospitalization as the primary outcome. In the study, 140 patients were treated with minocycline while the others were treated with a placebo. There were no statistical differences between these two groups regarding mortality. However, the drug showed a positive trend in patients older than 12 years, patients who survived the first day of hospitalization, and the Glasgow outcome score at three months [12]. The study by Kumar et al. had some problems. The population was too small, and the setting was in one of India's poorest regions, which compromised the study because of the lack of resources in the region [12].

In the Singh et al. study, 44 patients between ages one and 13 were given either minocycline or placebo in a placebo randomized controlled trial. Total hospital stay, duration of symptoms, and fever improved in the treatment group [13].

*Ribavirin*

Ribavirin is a broad-spectrum antiviral agent that can interfere with viral replication by inhibiting ribonucleic acid (RNA) and deoxyribonucleic acid (DNA) synthesis [14]. Up to now, there is no specific antiviral therapy that has proven effective in addressing the high mortality rate depicted in Japanese encephalitis. In a study to examine the effectiveness of oral ribavirin in addressing JE in children, no significant statistical differences between the drug and placebo groups were found, indicating that ribavirin is ineffective in reducing the early mortality associated with JE [14]. Perhaps the study obtained a negative outcome as a result of a significantly small study sample.

In a case report, a 52-year-old male had Japanese encephalitis that resembled an ischemic stroke. He was initially treated as if he were having a stroke. After nine days, he was diagnosed with JE and treated with ribavirin, and his symptoms improved. After six months of follow-up, he seemed normal with the exception of Parkinsonian features, especially when walking [7].

Immunoglobulin

Findings from in-vitro studies and animal models suggest that intravenous immunoglobulin (IVIG) with virus-specific neutralizing antibodies may be an effective treatment for JE [15].

In a study conducted in Nepal by Rayamajhi et al., the researchers performed a randomized, double-blind placebo-controlled IVIG trial [15]. In the study, 22 children were tested and 13 were confirmed to have JE; 11 received IVIG while 11 received a placebo. The adverse reactions were similar between both groups. There were no statistically significant differences in exhibiting full recovery in both groups. Neutralizing antibody titers were significantly high in the treatment group; the anti-Japanese encephalitis virus (anti-JEV) IgM titers were over 16 fold in the treatment group. There were also more levels of IL-4 and IL-6 in patients treated with IVIG [15]. However, the author argues that higher doses of IVIG could generate more neutralizing antibodies, improving patient outcomes with JE [15]. Future studies should consider using higher doses of IVIG.

Interferon

INF-α is produced as a natural response to the infection, as part of the innate immune system, and has been found to have antiviral activity against JEV [15].

Interferon alfa-2a was tested on patients with JE in a controlled randomized trial in 112 Vietnamese children. The result was the death of 21 children, and 17 had severe sequelae [16]. The researchers concluded that interferon alfa-2a is not effective in improving the outcomes of JE. The author suggested that given interferon at higher doses or with a different route might show a beneficial outcome if futures studies are conducted [16]. The children in the treatment group took slightly longer to talk, sit without support, walk independently, and leave the hospital than those in the control group [16].

Harinasuta et al. researched the effectiveness of interferon-alpha on two cases of JE in Thailand. The first case improved on the fifth day while the second case showed improvement on the sixth day [17]. The two patients in the control group died on the seventh and ninth day of illness. In this case series, interferon was shown to be an effective agent in treating JE; however, further studies on interferon-alpha-alpha are necessary [17].

Acyclovir

The use of acyclovir has only been reported in a case report and during an outbreak in Japan that affected six patients.

A 30-year-old man with flaccid paralysis due to JE was treated with acyclovir. The man received acyclovir for 12 days without any improvement. The patient did not die but had a number of neurological sequelae [6].

During an outbreak in Japan, six patients presented with Japanese encephalitis. All patients were treated with acyclovir. One patient died, four patients had neurological sequelae, and one patient did not have neurological sequelae at all. Further studies with placebo to compare should be done in the future. However, the evidence of acyclovir for the treatment of JE is not promising [18].

## Conclusions

JE is a debilitating infection that leaves a high percentage of those who suffer from the symptomatic disease with neurologic sequelae. This prompts researchers to investigate possible solutions to avoid the high morbidity and mortality rates accompanying the infection. Dexamethasone did not make statistically significant improvements to support its use. While minocycline showed efficacy in vitro and animal studies, trials on humans were not conclusive in one study, but the second study showed a statistically significant difference. In the case of ribavirin, the studies did not show beneficial outcomes, but the sample of the study was small, reducing the power of the study. Clinical findings did not support the use of IVIG as a treatment approach. Interferon use has contradictory results between studies and is a matter of further investigation. Finally, acyclovir did not improve the outcome of patients during the JE outbreak.

Among all drugs, minocycline showed the most promising results, with one study showing statistically significant results and another study showed a positive trend. The IVIG did not improve patients' outcomes but increase the level of neutralizing antibodies, IL-4, and IL-6; further studies with higher doses might change the outcomes in patients with JE. Overall, the other drugs failed to show promising results. In general, more studies should be done on most of the drugs discussed in this review. The need for definitive treatment and guidelines prompts the urgency of more investigation in this field.

## References

[1] Turtle L, Solomon T (2018). Japanese encephalitis - the prospects for new treatments. Nat Rev Neurol.

[2] Kari K, Liu W, Gautama K (2006). A hospital-based surveillance for Japanese encephalitis in Bali, Indonesia. BMC Med.

[3] Mansfield KL, Hernández-Triana LM, Banyard AC, Fooks AR, Johnson N (2017). Japanese encephalitis virus infection, diagnosis and control in domestic animals. Vet Microbiol.

[4] Misra UK, Kalita J (2010). Overview: Japanese encephalitis. Prog Neurobiol.

[5] Hoke CH Jr, Vaughn DW, Nisalak A (1992). Effect of high-dose dexamethasone on the outcome of acute encephalitis due to Japanese encephalitis virus. J Infect Dis.

[6] Shen Q, Li Y, Lu H, Ning P, Huang H, Zhao Q, Xu Y (2020). Acute flaccid paralysis as the initial manifestation of Japanese encephalitis: a case report. Jpn J Infect Dis.

[7] Zhao J, Chen F, Lu L, Li C, Du Y (2020). Japanese encephalitis (JE) mimicking acute ischemic stroke. A case report. Medicine (Baltimore).

[8] McNaughton H, Singh A, Khan SA (2018). An outbreak of Japanese encephalitis in a non-endemic region of north-east India. J R Coll Physicians Edinb.

[9] Fraley CE, Pettersson DR, Nolt D (2021). Encephalitis in previously healthy children. Pediatr Rev.

[10] Johnson RT, Intralawan P, Puapanwatton S (1986). Japanese encephalitis: identification of inflammatory cells in cerebrospinal fluid. Ann Neurol.

[11] Rathi AK, Kushwaha KP, Singh YD, Singh J, Sirohi R, Singh RK, Singh UK (1993). JE virus encephalitis: 1988 epidemic at Gorakhpur. Indian Pediatr.

[12] Kumar R, Basu A, Sinha S (2016). Role of oral Minocycline in acute encephalitis syndrome in India - a randomized controlled trial. BMC Infect Dis.

[13] Singh DAK, Mehta DA, Kushwaha DKP, Pandey AK, Mittal DM, Sharma DB, Pandey DJ (2016). Minocycline trial in Japanese encephalitis: a double blind, randomized placebo study. Ped Rev: Int J Ped Rev.

[14] Kumar R, Tripathi P, Baranwal M, Singh S, Tripathi S, Banerjee G (2009). Randomized, controlled trial of oral ribavirin for Japanese encephalitis in children in Uttar Pradesh, India. Clin Infect Dis.

[15] Rayamajhi A, Nightingale S, Bhatta NK (2015). A preliminary randomized double blind placebo-controlled trial of intravenous immunoglobulin for Japanese encephalitis in Nepal. PLoS One.

[16] Solomon T, Dung NM, Wills B (2003). Interferon alfa-2a in Japanese encephalitis: a randomized double-blind placebo-controlled trial. Lancet.

[17] Harinasuta C, Nimmanitya S, Titsyakorn U (1985). The effect of interferon-alpha A on two cases of Japanese encephalitis in Thailand. Southeast Asian J Trop Med Public Health.

[18] Ayukawa R, Fujimoto H, Ayabe M (2004). An unexpected outbreak of Japanese encephalitis in the Chugoku district of Japan, 2002. Jpn J Infect Dis.

